# Investigating Polymer Flipping and Lattice Disruptions in TELSAM-Facilitated Protein Crystallization

**DOI:** 10.1063/4.0001141

**Published:** 2025-10-27

**Authors:** Ethan Noakes, MJ Pedroza Romo, Joseph Gonzalez, Alihi Keliiliki, Eli Anderson, James D Moody

**Affiliations:** Brigham Young University, Provo, Utah, USA

## Abstract

TELSAM, the sterile alpha motif (SAM) domain of the human translocation ETS leukemia protein (TEL), spontaneously forms 6-fold helical polymers at low pH. Previously, TELSAM was fused to the CMG2 vWA domain via a Threonine–Valine linker to create the 1TEL-TV-vWA construct. This construct was crystallized, mounted in-house, and analyzed using synchrotron X-ray diffraction. Despite successful crystallization, structure solution consistently yielded high R-values. Reciprocal lattice analysis revealed three domains, indicating a break in lattice periodicity. The observed intensity pattern—normal reflections at h-k=3n and streaky reflections at h-k=3n±1—suggests a unit cell shift equivalent to one-third of the P6_5_ unit cell’s h-k length. This aligns with the polymer packing seen in the solved structure, where regular crystals show a repeating pattern of two normal polymers followed by a flipped one. The disrupted lattice shows a shift due to sequences of three or four normal polymers before the next flipped polymer. These flips occur because TELSAM polymers form crystal contacts independent of orientation, a known behavior in TELSAM-mediated crystallization. To address this lattice disruption and improve crystal quality, we have developed a novel device aimed at reducing polymer flipping during crystallization.

